# Direct borylation of terrylene and quaterrylene

**DOI:** 10.3762/bjoc.16.58

**Published:** 2020-04-06

**Authors:** Haruka Kano, Keiji Uehara, Kyohei Matsuo, Hironobu Hayashi, Hiroko Yamada, Naoki Aratani

**Affiliations:** 1 Division of Materials Science, Nara Institute of Science and Technology (NAIST), 8916-5 Takayama-cho, Ikoma 630-0192, Japan

**Keywords:** borylation, π-conjugation, oligorylene, single crystal X-ray structure, solubility

## Abstract

The preparation of large rylenes often needs the use of solubilizing groups along the rylene backbone, and all the substituents of the terrylenes and quaterrylenes were introduced before creating the rylene skeleton. In this work, we successfully synthesized 2,5,10,13-tetrakis(4,4,5,5-tetramethyl-1,3,2-dioxaborolan-2-yl)terrylene (**TB4**) by using an iridium-catalyzed direct borylation of C–H bonds in terrylene in 56% yield. The product is soluble in common organic solvents and could be purified without column chromatography. Single crystal X-ray diffraction analysis revealed that the terrylene core is not disturbed by the substituents and is perfectly flat. The photophysical properties of **TB4** are also unchanged by the substituents because the carbon atoms at 2,5,10,13-positions have less coefficients on its HOMO and LUMO, estimated by theoretical calculations. Finally, the same borylation reaction was applied for quaterrylene, resulting in the formation of soluble tetra-borylated quaterrylene despite a low yield. The post modification of rylenes enables us to prepare their borylated products as versatile units after creating the rylene skeletons.

## Introduction

**Figure 1:**
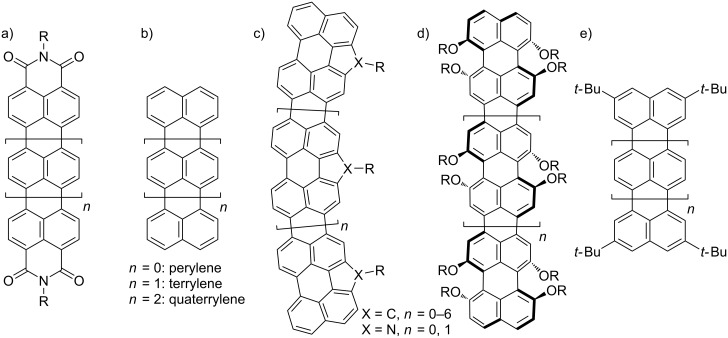
Chemical structures of a) oligorylene-bisimides, b) oligorylenes, c) bay-bridging oligorylenes, d) bay-alkoxy oligorylenes, and e) tetra-*tert*-butyl oligorylenes.

Compared with fruitful researches of oligorylene-bisimides for organic devices and single molecular spectroscopy [1–6], genuine oligorylenes have been sporadically investigated mainly because of their synthetic difficulty and low solubility (Figure 1) [7]. To be applied to electronic or photovoltaic components, the scalable synthesis of pure soluble compounds is essential. Recently, facile preparation methods of terrylene [8] and quaterrylene [9] were reported, after 50 years from the first reports, respectively [10–11]. Friedel–Crafts ring condensation reaction of 3-(1-naphthyl)perylene with AlCl_3_ reproducibly provided a pure terrylene [8]. Scholl reaction using a superacid catalyst in combination with 2,3-dichloro-5,6-dicyano-1,4-benzoquinone (DDQ) as oxidant provides a scalable preparation of quaterrylene [9], but unfortunately the low solubility prevents ^1^H NMR characterization. Larger oligorylenes have not been synthesized without the use of solubilizing groups along the rylene backbone.

For instance, bay-bridging perylenes with long alkyl chains [12–15] and bay-alkoxy-substitution [16–18] enable us to prepare longer rylenes (Figure 1c and 1d). However, tying the bay regions with carbon or nitrogen atoms causes bowing of the oligorylene backbone without disrupting the planarity of the aromatic backbone to give a flat banana-shaped molecule [15]. On the other hand, the bay-alkoxy-substitution is estimated to twist the core, which changes the original characteristics of rylenes but eventually helps to enhance the solubility of the macromolecules [7]. In order to avoid such deformation of the molecular skeleton, the solubilizing groups should be attained at the terminal positions. In this regard, the tetra-*tert*-butyl series of rylenes made it possible to study their intrinsic physical properties as a function of molecular length (Figure 1e) [19–20], while the low solubility of *tert*-butylpentarylene indicates that even such bulky group had reached its limit to interrupt π-stacking. The synthesis of higher oligorylenes would require more robust solubilizing groups.

All the substituents of the terrylenes and quaterrylenes were installed before creating the rylene skeleton and no derivatives have been produced by the post modification probably because their low solubility hampers such strategy. Therefore, the substituents on the rylene skeleton have been quite limited because they must suffer the harsh conditions of the rylene skeleton preparation.

Basic reactivity of rylenes with electrophiles is predicted from perylene in which the most reactive sites are the 3,4,9,10-positions [21]. On the other hand, the regioselectivity of the iridium (Ir)-catalyzed direct C–H borylation [22–25] gives 2,5,8,11-tetraborylated perylene [26]. The regioselectivity of the perylene borylation is determined by the steric factors rather than by the electron distribution in the arene and this regioselectivity complements that of electrophilic substitutions [26].

In light of the above, we plan to perform the Ir-catalyzed direct borylation of C–H bonds in terrylene because 1) the regioselectivity of the reaction is unique and should be remarkably high, thus terrylene will be borylated at the 2, 5, 10, and 13 positions, which don’t deform the rylene cores, 2) the product obtained is soluble in common organic solvents so that the separation and characterization would be readily performed, and 3) the resultant pure soluble terrylene derivative as a versatile unit can be functionalized by using conventional metal-catalyzed coupling reactions.

## Results and Discussion

According to the literature [8], terrylene was prepared from a 3-(1-naphthyl)perylene by using 8 equivalents of AlCl_3_ in chlorobenzene at 80 °C for 4 h. The crude product was purified by Soxhlet extraction followed by recrystallization from toluene. After these simple manipulations, the pure terrylene was isolated. Note that the terrylene is slightly soluble in halogenated solvents, hence a ^1^H NMR spectrum in CDCl_3_ at room temperature can be measured.

**Scheme 1:**
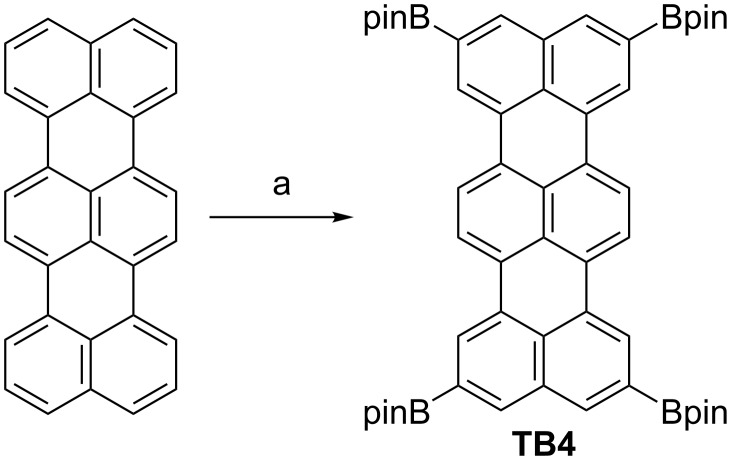
Synthesis of 2,5,10,13-tetrakis(4,4,5,5-tetramethyl-1,3,2-dioxaborolan-2-yl)terrylene (**TB4**): (a) (Bpin)_2_ (8 equiv), [Ir(OMe)cod]_2_ (10 mol %), di-*tert*-butylbipyridyl (20 mol %), 1,4-dioxane, at 105 °C, 30 h, yield 56%. Bpin = 4,4,5,5-tetramethyl-1,3,2-dioxaborolan-2-yl.

The reaction of terrylene (0.16 mmol), bis(pinacolato)diboron (8 equiv), [Ir(OMe)cod]_2_ (10 mol %) and di-*tert*-butylbipyridyl (20 mol %) in 1,4-dioxane (5 mL) under Ar at 105 °C for 30 h gave a deep orange solution (Scheme 1). Matrix assisted laser desorption/ionization (MALDI) mass spectrometry of the reaction mixture detected a single parent ion peak at *m*/*z* = 880.4687 (calcd for C_54_H_60_B_4_O_8_ = 880.4660 [M]^+^), making us expect its selective tetra-substitution with high yield. To isolate a pure product, the solvent was removed, then the reaction mixture was passed through a silica pad with CH_2_Cl_2_, followed by reprecipitation from MeOH. The yield of 2,5,10,13-tetrakis(4,4,5,5-tetramethyl-1,3,2-dioxaborolan-2-yl)terrylene (**TB4**) was 56%. Since insoluble materials and polar byproducts can be removed by short silica gel and the formation of tri-, bis- and mono-substituted compounds is negligible, **TB4** can be purified without column chromatography.

**Figure 2:**
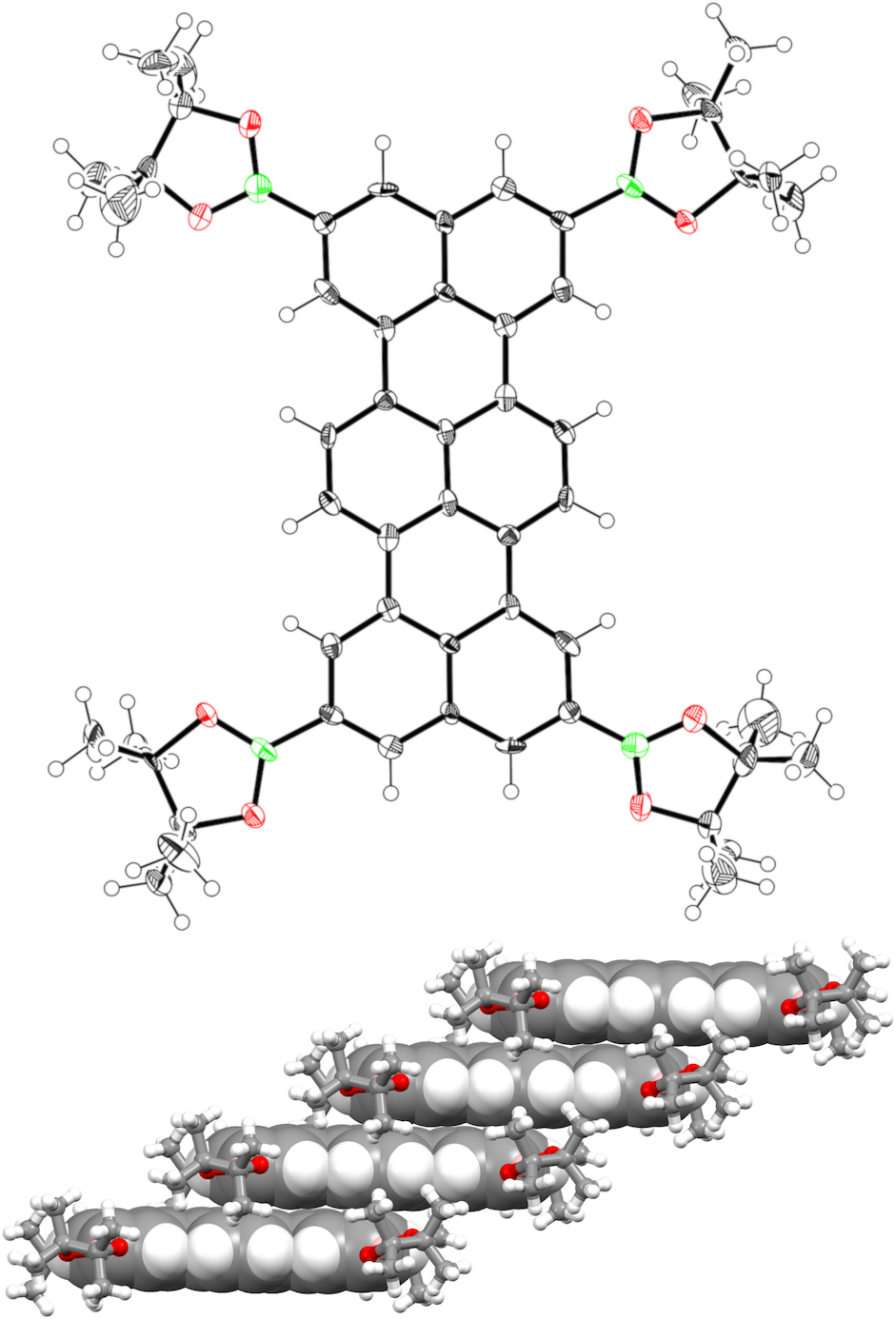
(Top) Single crystal X-ray structure of **TB4**. The thermal ellipsoids are scaled at 50% probability. (Bottom) Packing diagram of **TB4**. The solvent molecules are omitted for clarity.

The ^1^H NMR spectrum of **TB4** in CDCl_3_ exhibited a highly symmetric *D*_2_*_h_* signal pattern that consists of three singlet peaks at 8.58, 8.37 and 8.23 ppm due to aromatic protons. The structure of **TB4** was unambiguously revealed by single crystal X-ray diffraction analysis (Figure 2). The crystals suitable for X-ray diffraction were obtained by vapor diffusion of hexane into a solution of **TB4** in CH_2_Cl_2_. The terrylene core is perfectly planar and forms a face-to-face stacking columnar structure with an interplanar distance of 3.40 Å along the *a* axis. The BO_2_ units are slightly tilted from the aromatic plane (6.7 and 20.3°).

**Figure 3:**
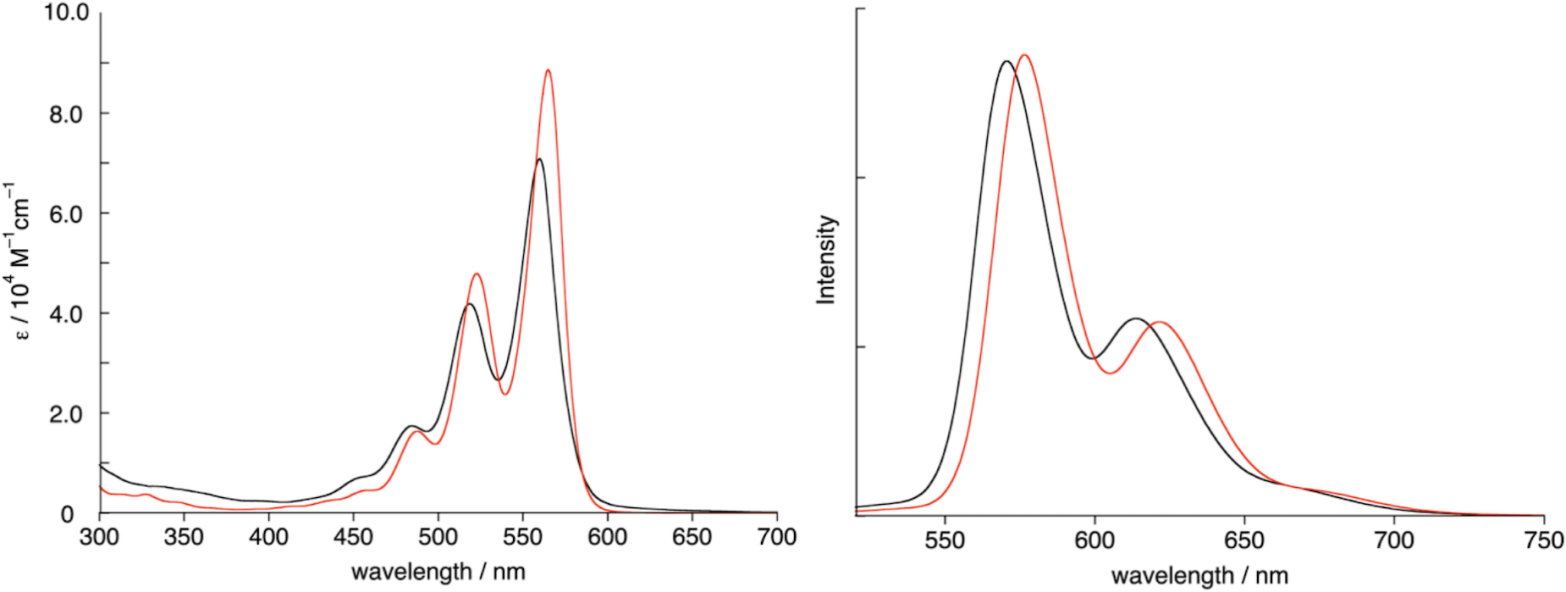
UV–vis absorption and fluorescence spectra of terrylene (black) and **TB4** (red) in toluene. λ_ex_ = 489 nm. Fluorescence intensity reflects the absolute fluorescence quantum yields.

**TB4** in toluene absorbs UV–vis light with an absorption maximum at 566 nm, and emits fluorescence at 576 nm with a quantum yield of Φ_F_ = 0.86 at 298 K (Figure 3). Both peaks are slightly red-shifted relative to those of intact terrylene (λ_abs_ = 560 nm and λ_em_ = 571 nm with Φ_F_ = 0.82 in toluene).

**Figure 4:**
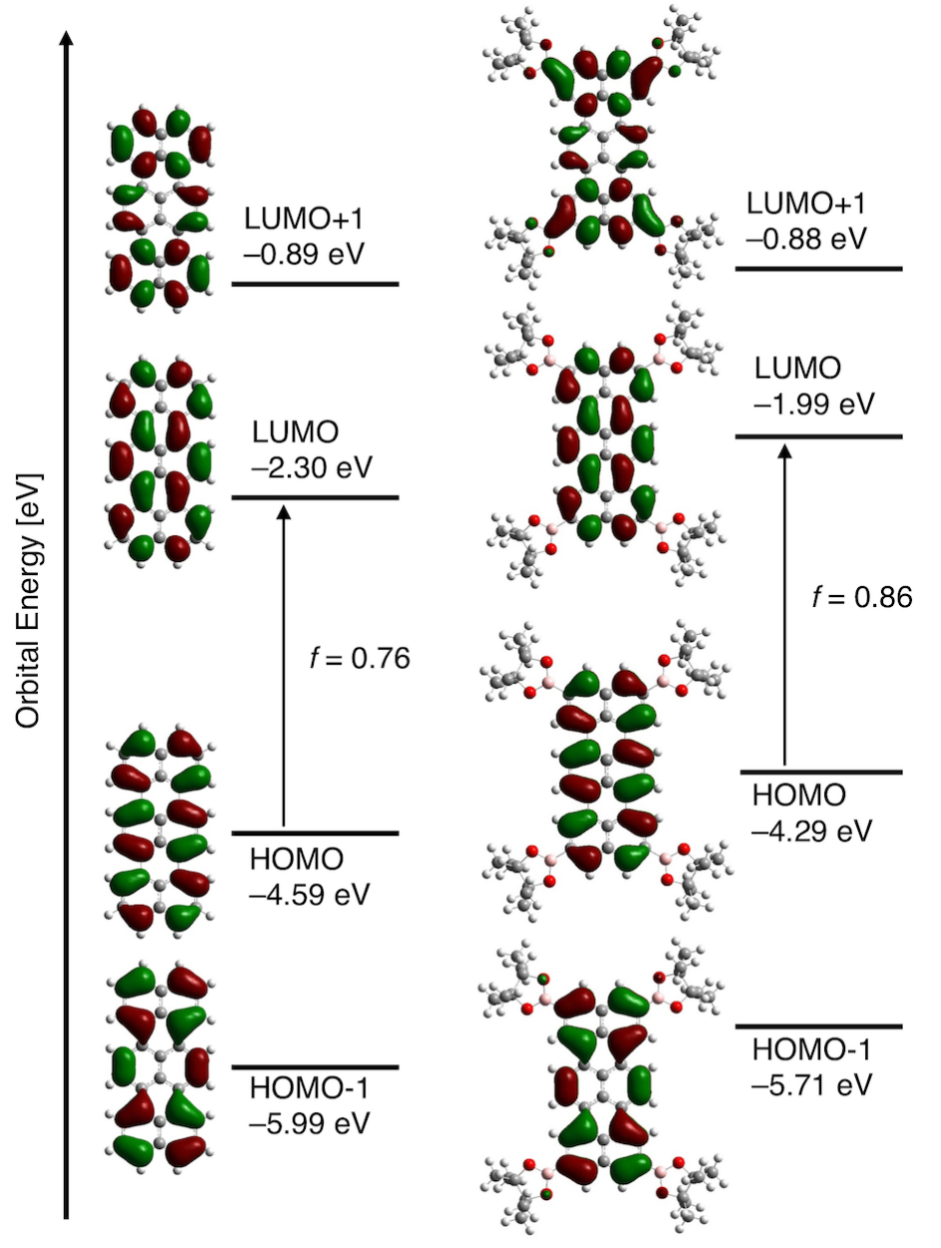
MO diagrams of terrylene and **TB4** based on calculations at the B3LYP/6-31G(d).

We employed density functional theory (DFT) and time-dependent (TD)-DFT calculations, both of them at the B3LYP/6-31G(d) level and with Gaussian 09, to make these electronic features understandable (Figure 4) [27]. The HOMO, HOMO–1, LUMO, and LUMO+1 for terrylene are nondegenerative and the coefficient distribution in these four frontier MOs seem to be delocalized over the whole aromatic core. The highest band of terrylene at 553 nm is mainly caused by transition from HOMO (−4.59 eV) to LUMO (−2.30 eV) (oscillator strength, *f* = 0.76). Although the frontier MOs for **TB4** are overall destabilized due to the smaller electronegativity of boron, the impact of substituents to the HOMO and LUMO distributions is negligible because of the existence of node at 2,5,10,13-positions. The longest band of **TB4** at 563 nm comprises the transition from equally destabilized HOMO (−4.29 eV) to LUMO (−1.99 eV) (oscillator strength, *f* = 0.86). The transition energies and oscillator strengths simulated by TD-DFT calculations showed a good agreement with the observed absorption spectra.

**Scheme 2:**
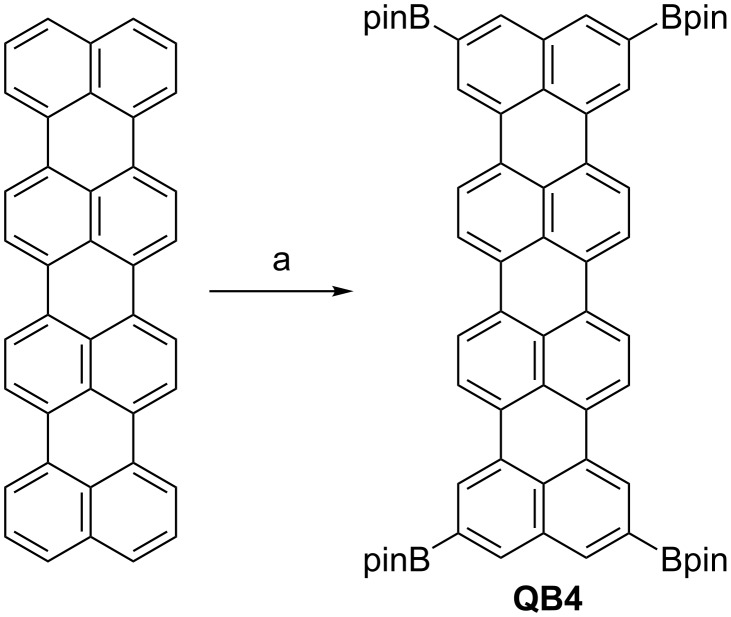
Synthesis of 2,5,12,15-tetrakis(4,4,5,5-tetramethyl-1,3,2-dioxaborolan-2-yl)quaterrylene (**QB4**): (a) (Bpin)_2_ (12 equiv), [Ir(OMe)cod]_2_ (20 mol %), di-*tert*-butylbipyridyl (40 mol %), 1,4-dioxane, at 105 °C, 38 h, yield 0.4%.

Taking the successful result of the terrylene borylation, next we tried to perform the same reaction to quaterrylene (Scheme 2). The quaterrylene was prepared by the oxidative condensation reaction of perylene with TfOH and DDQ [9]. However, the crude product was not completely purified by Soxhlet extraction and by crystallization in our hands. The borylation reaction of hardly soluble crude quaterrylene gave a deep green suspension. MALDI mass spectrometry of the reaction mixture detected an ion peak at *m*/*z* = 1004.5018 (calcd for C_64_H_64_B_4_O_8_ = 1004.5003 [M]^+^), indicating that the mixture contains tetrasubstituted quaterrylene. To isolate the product, the combination of silica gel column chromatography and gel permeation chromatography techniques, followed by reprecipitation from MeOH gave a 2,5,12,15-tetrakis(4,4,5,5-tetramethyl-1,3,2-dioxaborolan-2-yl)quaterrylene (**QB4**) in 0.4% yield. The poor solubility of starting material prevented a smooth reaction.

It is difficult to observe accurate characteristics of quaterrylene in solution owing to its remarkable insolubility. The much improved solubility of **QB4** enables ^1^H NMR measurement in CDCl_3_, which exhibits a highly symmetric *D*_2_*_h_* signal pattern that consists of two singlet peaks at 8.63 and 8.25 ppm, and two doublet peaks at 8.44 and 8.32 ppm for aromatic protons.

**Figure 5:**
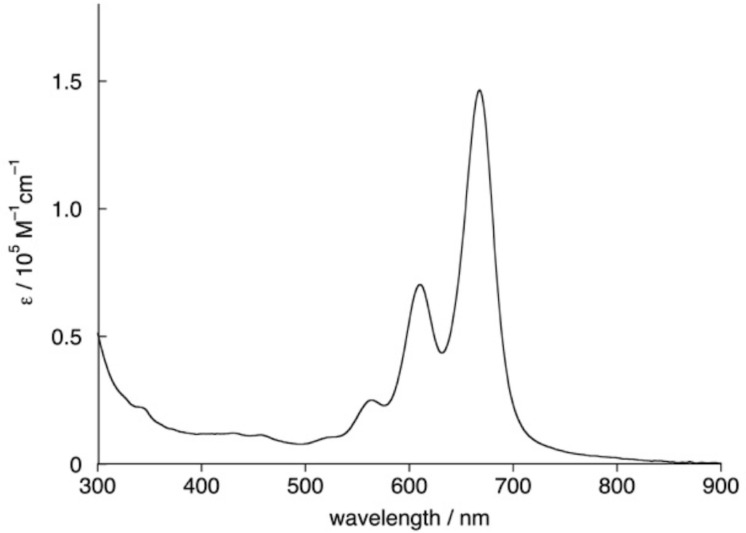
UV–vis absorption spectrum of **QB4** in toluene.

The UV–visible absorption spectrum of **QB4** in toluene is shown in Figure 5. Compared with *tert*-butylquaterrylene in dioxane [19], a slightly red-shifted absorption peak at 668 nm is observed. Interestingly, **QB4** emits only weak fluorescence as was described for tetra-*tert*-butylquaterrylene [20].

**Figure 6:**
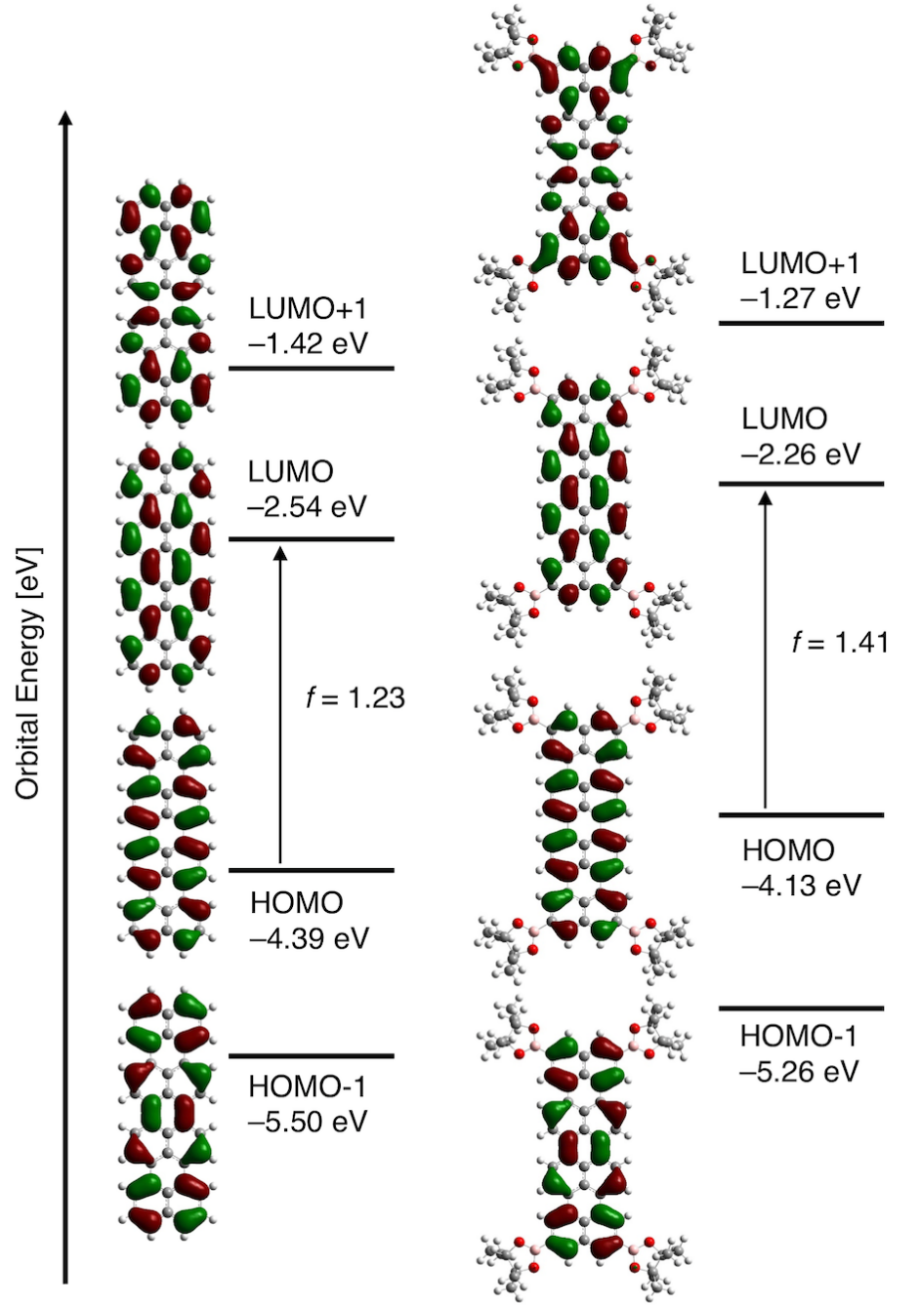
MO diagrams of terrylene and **QB4** based on calculations at the B3LYP/6-31G(d).

The coefficient distribution of four frontier MOs of quaterrylenes basically exhibits the same symmetric pattern with terrylenes (Figure 6). The longest band of **QB4** at 676 nm comprises the transition from high energy level of HOMO (−4.13 eV) to LUMO (−2.26 eV) (oscillator strength, *f* = 1.41). The simulated absorption showed a good agreement with the observed spectra.

The calculated HOMO levels of –4.29 eV for **TB4** and –4.13 eV for **QB4** are pretty high among electron-rich compounds. Thus we tested the stability of **TB4** and **QB4** under air by means of UV–vis absorption spectroscopy. Surprisingly almost no spectral changes for both compounds were observed after 12 h, probably because the most reactive sites are protected by the steric hinderance of Bpin moieties.

**Scheme 3:**
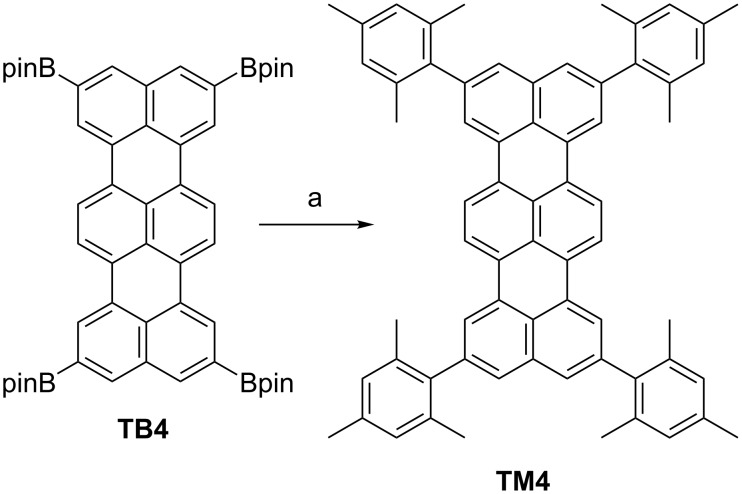
Suzuki–Miyaura cross-coupling reaction of **TB4** with 2-bromomesitylene. (a) 2-bromomesitylene (8 equiv), Pd(PPh_3_)_4_ (40mol %), Cs_2_CO_3_ (40 equiv), CsF (40 equiv), toluene/DMF (1:1), at 110 °C, 24 h, yield 58%.

Finally, to demonstrate the utility of the borylated oligorylenes, the Suzuki–Miyaura cross-coupling reaction of **TB4** under the standard conditions was performed (Scheme 3). Coupling of **TB4** and 2-bromomesitylene with Pd(PPh_3_)_4_, Cs_2_CO_3_ and CsF in a mixture of toluene/DMF furnished 2,5,10,13-tetramesitylterrylene (**TM4**) in 58% yield. **TM4** was successfully isolated through a silica gel pad and by reprecipitation. The structure of **TM4** was characterized by mass spectrometry and ^1^H and ^13^C NMR spectroscopy. High-resolution MALDI mass spectrometry detected the parent ion peak at *m*/*z* = 848.4377 (calcd. for C_66_H_56_ = 848.4377 [M]^+^). The ^1^H NMR spectrum of **TM4** in CDCl_3_ reveals only a single set of signals that consists of four singlet peaks for aromatic protons at 8.21, 8.02, 7.44, and 7.03 ppm.

## Conclusion

In this work, we synthesized soluble terrylene and quaterrylene derivatives by using an Ir-catalyzed borylation reaction. Our results provide a highly potential route to various functional oligorylene derivatives. In fact, **TB4** could be transformed into the tetraarylterrylene **TM4** in good yield by the conventional cross-coupling reaction. **TB4** exhibits face-to-face interaction in the crystals, resulting in the 1D columnar structure as a whole, which is expected to have higher carrier mobility. Recently, oligo-*peri*naphthalenes, namely oligorylenes, are of great interest as good model compounds of armchair graphene nanoribbons (AGNRs), since AGNRs with a width of *N* = 3*p* + 2 are predicted to be metallic with a small bandgap [28–30], and the narrowest AGNR is polyrylene (5-AGNR, *p* = 1) [31–32]. Many potential applications await techniques to prepare large scale quantities of functionalized oligorylenes. We believe that our approach opens fruitful oligorylene researches that find many uses in organic devices.

## Experimental

^1^H NMR (500 MHz) and ^13^C NMR (126 MHz) spectra were recorded with a JEOL JNM-ECX500 spectrometer at 20 °C by using tetramethylsilane as an internal standard. High-resolution mass spectra (HRMS) were measured with the matrix-assisted laser desorption/ionization-time-of-flight (MALDI–TOF) method on a JEOL SpiralTOF/JMS-S3000 spectrometer. X-ray crystallographic data were recorded at 103 K with a BRUKER-APEXII X-ray diffractometer using Mo Kα radiation equipped with a large area CCD detector. UV–vis absorption spectra were measured with a JASCO UV/Vis/NIR spectrophotometer V-570, and fluorescence spectra were measured with a JASCO PL spectrofluorometer FP-6600. Fluorescence quantum yields were measured on a HAMAMATSU Absolute PL Quantum Yield Measurement System C9920-02G. TLC and gravity column chromatography were performed on Art. 5554 (Merck KGaA) plates and silica gel 60N (Kanto Chemical), respectively. All solvents and chemicals were of reagent-grade quality, obtained commercially, and used without further purification. Terrylene and quaterrylene were prepared according to the literature [8–9]. For spectral measurements, spectral-grade toluene was purchased from Nacalai Tesque.

Synthesis of **TB4**. [Ir(OMe)(cod)]_2_ (10.6 mg, 16 μmol), di-*tert*-butylbipyridyl (8.6 mg, 32 μmol), terrylene (60 mg, 0.16 mmol) and (Bpin)_2_ (325 mg, 1.3 mmol) were added to a flask and dissolved in 1,4-dioxane (5 mL) under Ar. After bubbled with Ar for 10 min, the mixture was heated at 105 °C for 30 h and concentrated. Extracted with CH_2_Cl_2_ and washed with H_2_O, dried over Na_2_SO_4_ and concentrated. The residue was dissolved with CH_2_Cl_2_ and passed through a pad of silica gel and reprecipitated with MeOH to afford **TB4** (78 mg, 56%). ^1^H NMR (500 MHz, CDCl_3_) δ 8.58 (s, 4H), 8.37 (s, 4H), 8.23 (s, 4H), 1.44 (s, 48H) ppm; ^13^C NMR (126 MHz, CDCl_3_) δ 136.60, 133.49, 131.50, 130.71, 130.53, 129.88, 127.02, 126.04, 121.61, 84.61, 25.28 ppm; HRMS (Spiral MALDI) *m*/*z*: [M]^+^ calcd for C_54_H_60_B_4_O_8_, 880.4686; found, 880.4687; UV–vis (toluene): λ_max_ (ε [10^4^ M^−1^ cm^−1^]) = 489 (1.5), 524 (4.1) and 566 (7.2) nm; fluorescence (toluene): λ_max_ (λ_ex_ = 489 nm) = 576 and 622 nm.

Crystallographic data have been deposited with Cambridge Crystallographic Data Centre: Deposition number CCDC-1981465. Copies of the data can be obtained free of charge via http://www.ccdc.cam.ac.uk/conts/retrieving.html.

Synthesis of **QB4**. [Ir(OMe)(cod)]_2_ (26.5 mg, 0.04 mmol), di-*tert*-butylbipyridyl (21.5 mg, 0.08 mmol), quaterrylene (100 mg, 0.20 mmol) and (Bpin)_2_ (609 mg, 2.4 mmol) was added to a flask and dissolved in 1,4-dioxane (2 mL) under Ar. After bubbled with Ar for 10 min, the mixture was heated at 105 °C for 38 h and concentrated. Extracted with CH_2_Cl_2_ and washed with H_2_O, dried over Na_2_SO_4_ and concentrated. The residue was dissolved with CH_2_Cl_2_ and passed through a pad of silica gel. The combination of silica gel column chromatography and gel permeation column chromatography, followed by reprecipitation with MeOH afforded **QB4** (0.7 mg, 0.4%). ^1^H NMR (500 MHz, CDCl_3_) δ 8.63 (s, 4H), 8.44 (d, *J* = 8.5 Hz, 4H), 8.32 (d, *J* = 8.5 Hz, 4H), 8.25 (s, 4H), 1.44 (s, 48H) ppm; HRMS (Spiral MALDI) *m*/*z*: [M]^+^ calcd for C_64_H_64_B_4_O_8_, 1004.5003; found, 1004.5018; UV–vis (toluene): λ_max_ = 565, 610 and 668 nm.

Synthesis of **TM4**. **TB4** (8.8 mg, 0.01 mmol), 2-bromomesitylene (7.2 μL, 0.08 mmol), Pd(PPh_3_)_4_ (4.6 mg, 4 μmol), Cs_2_CO_3_ (130 mg, 0.4 mmol) and CsF (61 mg, 0.4 mmol) were added to a flask. The atmosphere was exchanged by applying vacuum and backfilling with Ar (this process was conducted three times). Toluene (0.3 mL) and DMF (0.3 mL) were added to the flask and the resulting mixture was stirred at 110 °C for 24 h. Upon cooling to room temperature, the mixture was extracted with Et_2_O, washed with H_2_O, and evaporated. The residue was dissolved with CH_2_Cl_2_ and passed through a pad of silica gel, followed by reprecipitation with MeOH to afford **TM4** (4.9 mg, 58%). ^1^H NMR (500 MHz, CDCl_3_) δ 8.21 (s, 4H), 8.02 (s, 4H), 7.44 (s, 4H), 7.03 (s, 4H), 2.38 (s, 12H), 2.16 (s, 24H) ppm; ^13^C NMR (126 MHz, CDCl_3_) δ 139.83, 138.83, 137.13, 136.34, 135.49, 131.51, 130.83, 129.90, 128.35, 127.90, 126.22, 122.10, 121.59, 21.26, 21.15 ppm; HRMS (Spiral MALDI) *m*/*z*: [M]^+^ calcd for C_66_H_56_, 848.4377; found, 848.4377; UV–vis (toluene): λ_max_ (ε [10^4^ M^−1^ cm^−1^]) = 493 (1.8), 528 (4.9) and 572 (8.6) nm; fluorescence (toluene): λ_max_ (λ_ex_ = 489 nm) = 583, 629 and 682 nm.

## Supporting Information

File 1 Crystallographic information file (cif) for the **TB4** crystal.

File 2 ^1^H and ^13^C NMR as well as MS spectra for newly synthesized compounds.
