# Supplementary material for: Direct borylation of terrylene and quaterrylene
Source: Beilstein J Org Chem. 2020 Apr 6;16:621–7. doi: 10.3762/bjoc.16.58 (PMC7155900; doi:10.3762/bjoc.16.58)
Supplement: File 2 — 1H and 13C NMR as well as MS spectra for newly synthesized compounds. [file Beilstein_J_Org_Chem-16-621-s002.pdf]

BEILSTEIN JOURNAL OF ORGANIC CHEMISTRY

Supporting Information

for

Direct borylation of terrylene and quaterrylene

Haruka Kano, Keiji Uehara, Kyohei Matsuo, Hironobu Hayashi, Hiroko Yamada and Naoki Aratani

Beilstein J. Org. Chem. **2020,** *16,* 621–627. doi:10.3762/bjoc.16.58

^{1}H and ^{13}C NMR as well as MS spectra for newly synthesized compounds

License and Terms: This is a supporting information file under the terms of the Creative Commons Attribution License (http://creativecommons.org/licenses/by/4.0). Please note that the reuse, redistribution and reproduction in particular requires that the authors and source are credited.
The license is subject to the *Beilstein Journal of Organic Chemistry* terms and conditions: (https://www.beilstein-journals.org/bjoc)

1. NMR Spectra

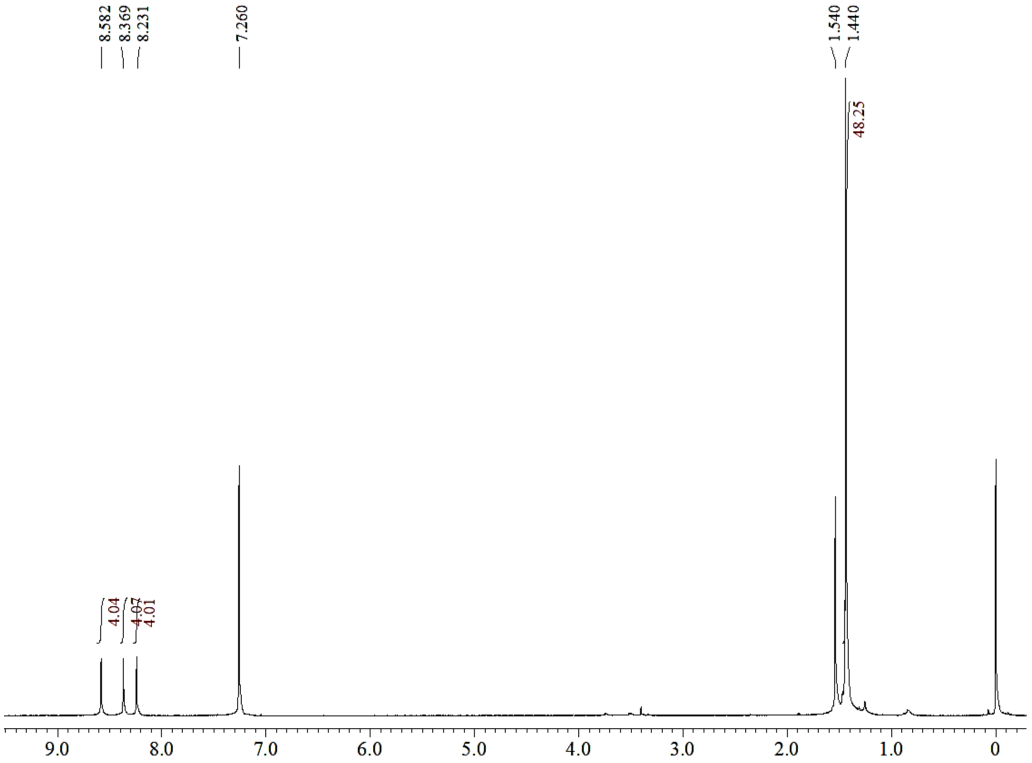

Figure S1. ^{1}H NMR spectrum of **TB4** in $CDCl_3$ at room temperature.

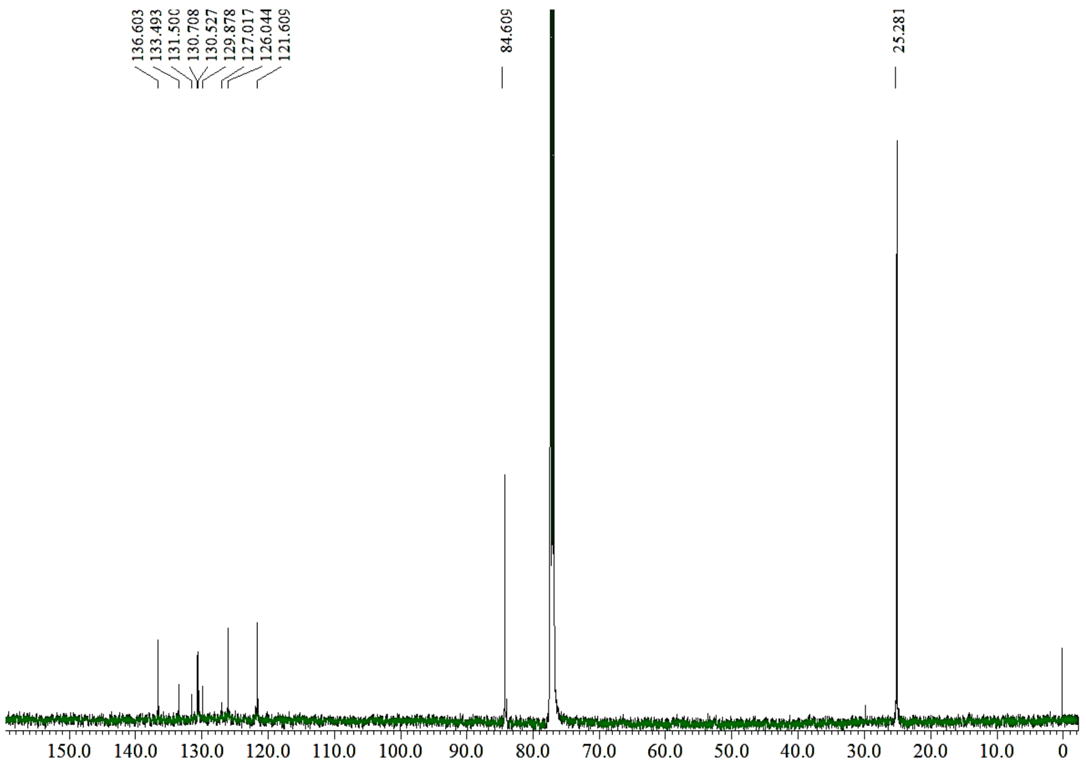

Figure S2. ^{13}C NMR spectrum of **TB4** in $CDCl_3$ at room temperature.

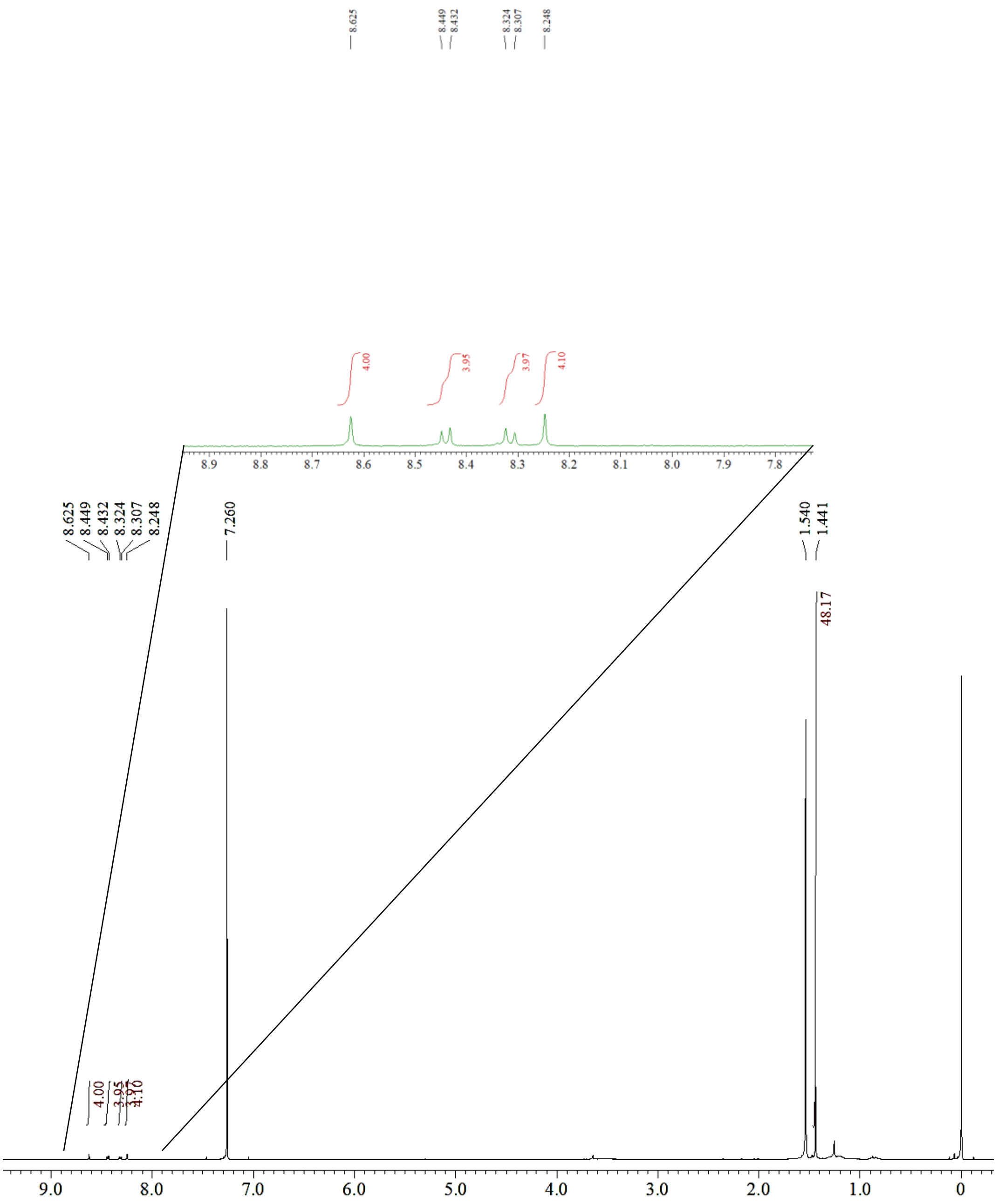

Figure S3. ^{1}H NMR spectrum of **QB4** in $CDCl_3$ at room temperature.

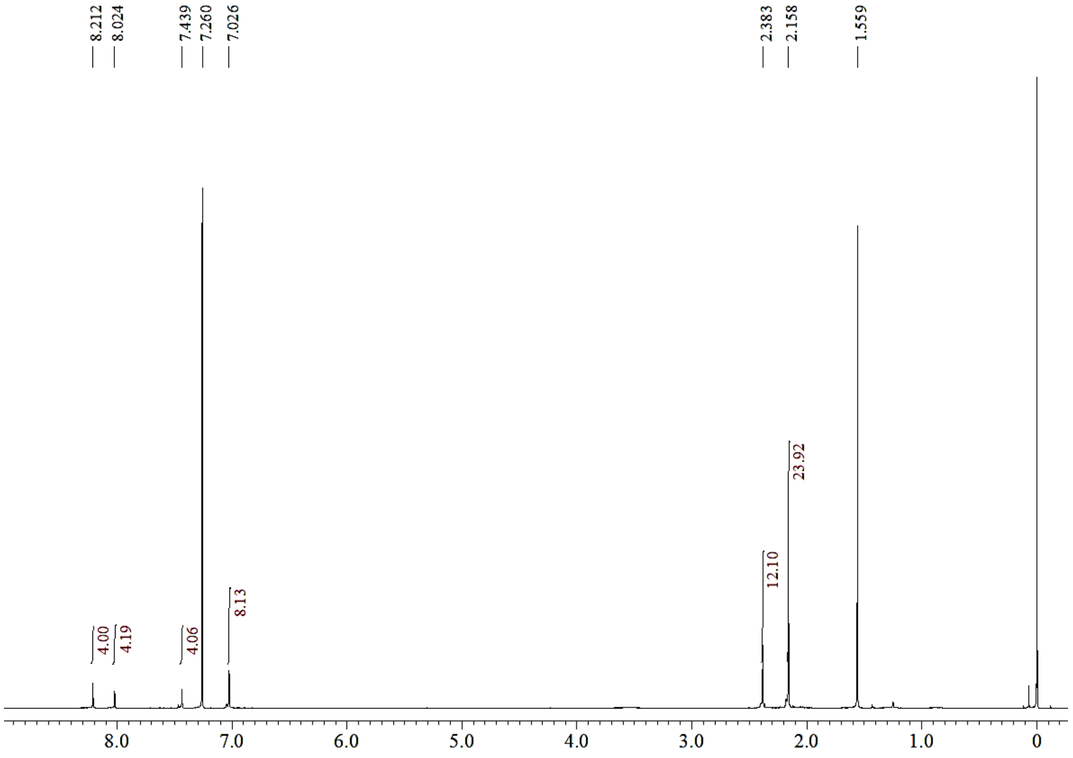

Figure S4. ^{1}H NMR spectrum of **TM4** in $CDCl_3$ at room temperature.

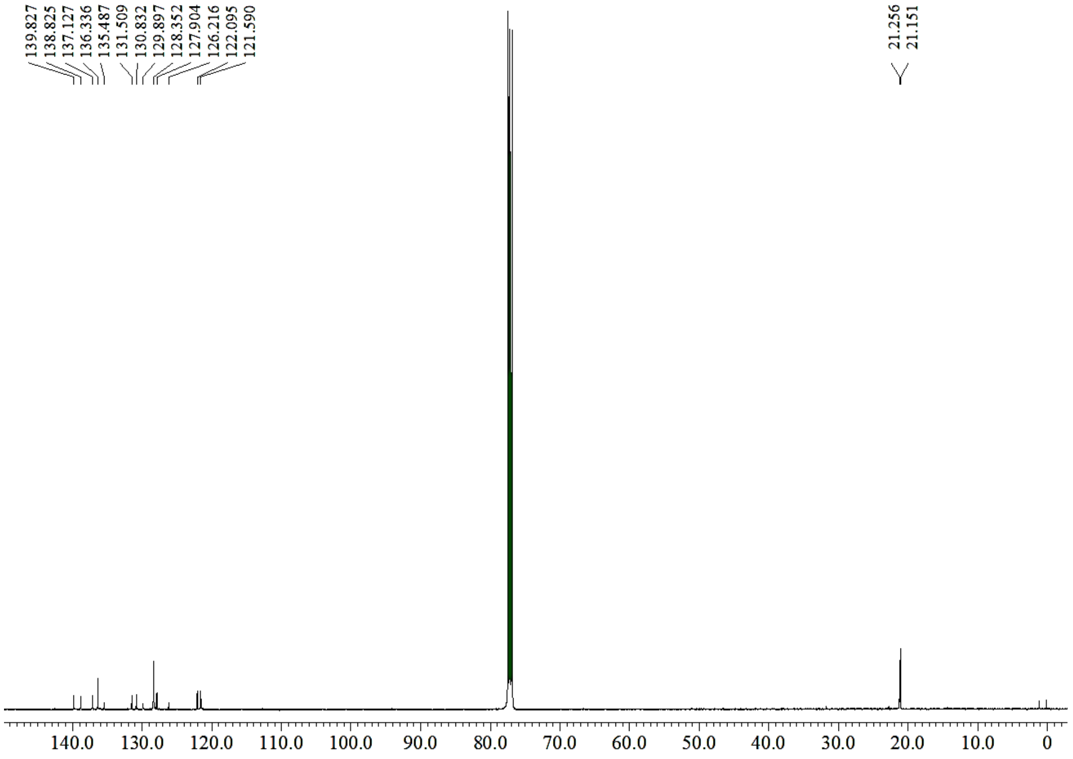

Figure S5. ^{13}C NMR spectrum of **TM4** in $CDCl_3$ at room temperature.

2. HR-MS

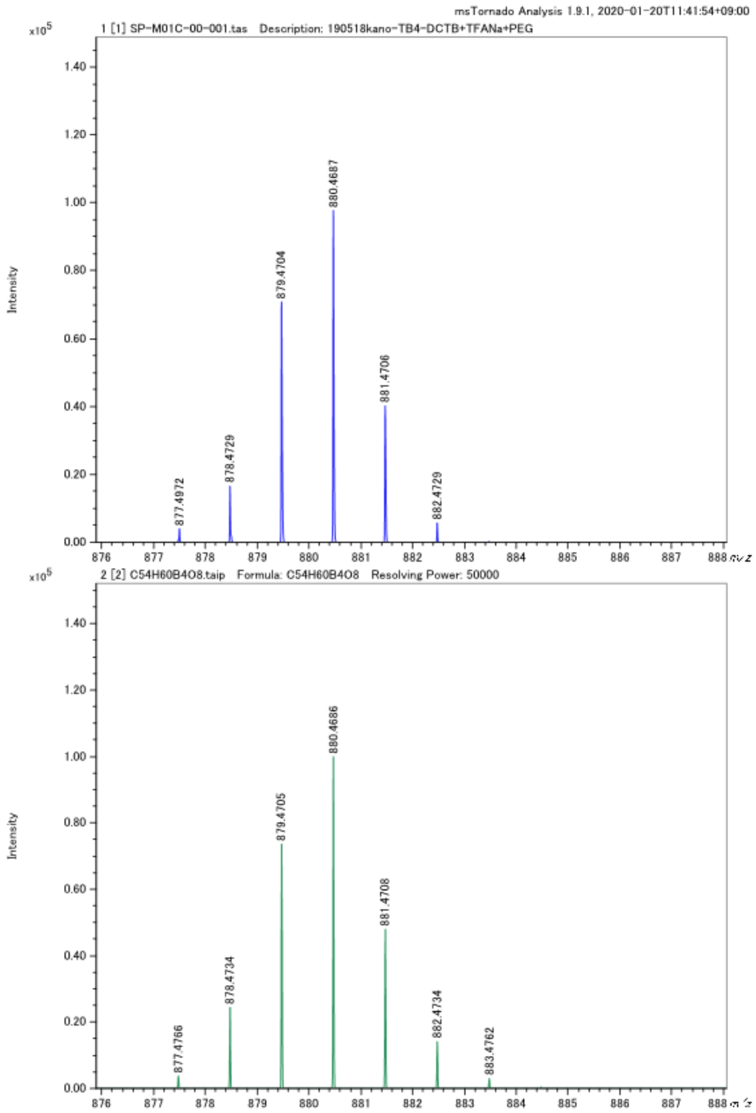

Figure S6. HR-Spiral-MALDI-TOF mass spectrum of **TB4**.

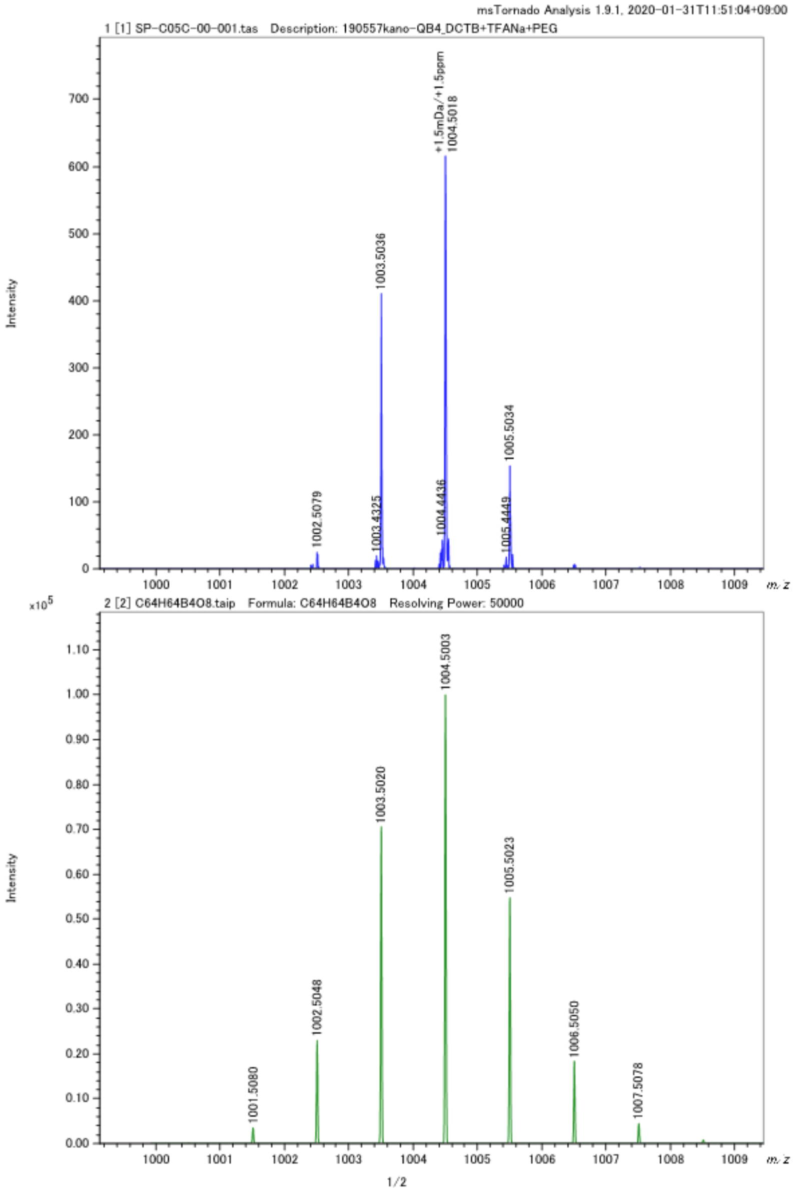

Figure S7. HR-Spiral-MALDI-TOF mass spectrum of **QB4**.

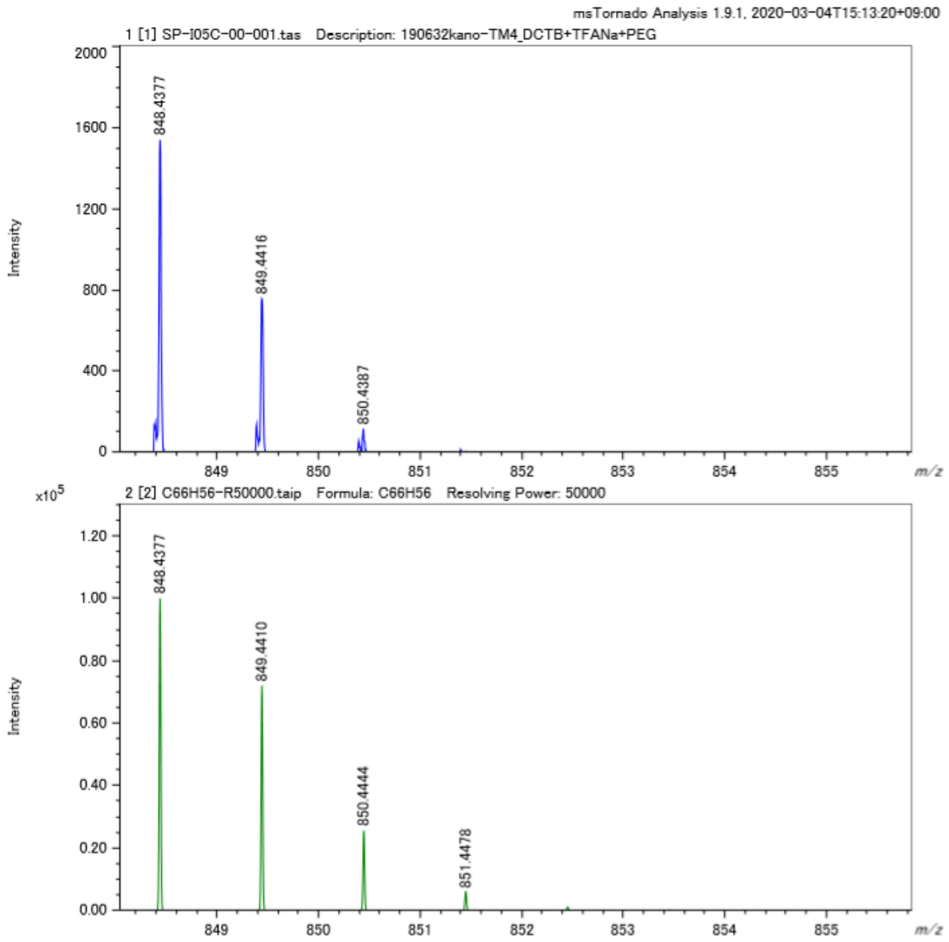

Figure S8. HR-Spiral-MALDI-TOF mass spectrum of **TM4**.

3. X-ray Crystal Structure

Table S1. Crystal data and structure refinement for **TB4**.

Empirical formula	$C_{56}H_{64}B_4Cl_4O_8$
Formula weight	1050.11
Temperature	103(2) K
Wavelength	0.71073 Å
Crystal system	monoclinic
Space group	$P2_1/c$
Unit cell dimensions	a = 6.691(5) Å
	b = 29.13(2) Å β = 97.183(17)°
	c = 14.044(12) Å
Volume	2716(4) Å^3
Z	2
Density (calculated)	1.284 g/cm^3
Absorption coefficient	0.271 mm^{-1}
F(000)	1104
Crystal size	0.150 x 0.020 x 0.010 mm^3
Theta range for data collection	1.620 to 23.619°
Index ranges	$-7 \le h \le 7$, $-30 \le k \le 31$, $-15 \le l \le 5$
Reflections collected	6825
Independent reflections	3943 [R(int) = 0.1642]
Completeness to theta = 23.619°	96.5%
Max. and min. transmission	0.997 and 0.817
Refinement method	Full-matrix least-squares on F^2
Data / restraints / parameters	3943 / 18 / 333
Goodness-of-fit on F^2	0.925
Final R indices [$I > 2\sigma(I)$]	R_1 = 0.0847, wR_2 = 0.1476
R indices (all data)	R_1 = 0.2590, wR_2 = 0.2254
Largest diff. peak and hole	0.435 and –0.323 e.Å^{-3}

4. Cartesian Coordinates

The cartesian coordinates (in Å) optimized at the B3LYP/6-31G(d) level are shown.

Terrylene

C	2.48025	-4.64431	-0.01703
C	1.25785	-3.92981	-0.01863
C	1.20705	-2.54161	-0.02883
C	2.44825	-1.82651	-0.02873
C	3.66675	-2.54041	-0.02613
C	3.67265	-3.96481	-0.02193
C	2.46425	-0.39421	-0.02813
C	3.70015	0.24039	-0.02813
C	4.92115	-0.47611	-0.02803
C	4.91165	-1.84851	-0.02643
C	-0.05835	-1.78991	-0.02773
C	-0.06265	-0.35691	-0.01563
C	1.18895	0.34099	-0.02433
C	-1.28685	-2.43111	-0.05703
C	-2.50265	-1.71941	-0.02223
C	-2.53945	-0.33601	0.04097
C	-1.28845	0.36039	-0.00193
C	-1.29715	1.79279	-0.03783
C	-0.07075	2.43739	-0.03243
C	1.14625	1.72619	-0.02413
C	-3.81155	0.39869	0.11957
C	-3.80195	1.82739	0.02567
C	-2.56445	2.54089	-0.07463
C	-5.03495	-0.23401	0.29887
C	-6.25725	0.48029	0.32997
C	-6.25915	1.84589	0.18827
C	-5.02225	2.53739	0.04107
C	-5.03405	3.95609	-0.08633
C	-3.84655	4.63259	-0.21433
C	-2.62135	3.92259	-0.20473
H	2.47421	-5.74428	-0.01186
H	0.31393	-4.49458	-0.01153
H	4.62487	-4.51552	-0.02261
H	3.73111	1.33996	-0.02821
H	5.8776	0.06721	-0.02923
H	5.85782	-2.40954	-0.02539

H	-1.3136	-3.52955	-0.10906
H	-3.44971	-2.27843	-0.04593
H	-0.04602	3.53711	-0.03468
H	2.09175	2.28832	-0.01728
H	-5.05503	-1.32704	0.42085
H	-7.20585	-0.05932	0.46775
H	-7.20833	2.40181	0.18839
H	-5.98721	4.50516	-0.08217
H	-3.84665	5.72707	-0.32444
H	-1.68136	4.48526	-0.30382

TB4

C	8.54731	0.2419	-0.06966
C	9.76831	-0.4746	-0.06956
C	9.75881	-1.847	-0.06796
C	8.51391	-2.5389	-0.06766
C	7.29541	-1.825	-0.07026
C	7.31141	-0.3927	-0.06966
C	8.51981	-3.9633	-0.06346
C	7.32741	-4.6428	-0.05856
C	6.10501	-3.9283	-0.06016
C	6.05421	-2.5401	-0.07036
C	6.03611	0.3425	-0.06586
C	4.78881	-1.7884	-0.06926
C	4.78451	-0.3554	-0.05716
C	3.55871	0.3619	-0.04346
C	3.55001	1.7943	-0.07936
C	4.77641	2.4389	-0.07396
C	5.99341	1.7277	-0.06566
C	3.56031	-2.4296	-0.09856
C	2.34451	-1.7179	-0.06376
C	2.30771	-0.3345	-0.00056
C	2.28271	2.5424	-0.11616
C	1.03561	0.4002	0.07804
C	1.04521	1.8289	-0.01586
C	-0.17509	2.5389	-0.00046
C	-0.18689	3.9576	-0.12786
C	1.00061	4.6341	-0.25586
C	2.22581	3.9241	-0.24626
C	-0.18779	-0.2325	0.25734

C	-1.41009	0.4818	0.28844
C	-1.41199	1.8474	0.14674
B	-2.78124	-0.29818	0.4876
B	1.00047	6.21611	-0.41503
O	-2.89842	-1.81747	0.65552
O	-4.16075	0.37046	0.53699
O	-0.16489	7.02999	-0.98924
O	2.16626	7.12874	-0.01427
C	-5.0565	-0.7482	0.46483
C	-4.25472	-1.97976	1.0934
C	-4.82298	-3.29863	0.58624
C	-4.35895	-2.00584	2.6126
C	-6.31964	-0.47034	1.26906
C	-5.49333	-1.03036	-0.96663
C	0.19375	8.37538	-0.64439
C	1.79098	8.38993	-0.58656
C	-0.30885	9.34771	-1.7034
C	-0.42376	8.80307	0.68049
C	2.28071	9.53695	0.28753
C	2.40418	8.58868	-1.96642
B	11.15082	0.31075	-0.0713
B	7.31869	-6.23276	-0.05108
O	11.28065	1.83826	-0.07315
O	12.53006	-0.36027	-0.07135
C	12.66954	2.03418	0.22722
C	13.4152	0.73759	-0.33631
C	13.19068	3.28865	-0.46145
C	12.90453	2.21696	1.72084
C	14.74332	0.53452	0.3809
C	13.72655	0.86612	-1.82159
O	6.86705	-7.10322	-1.22942
O	7.76103	-7.09745	1.13602
C	7.32423	-8.40805	-0.84729
C	7.3247	-8.40907	0.75106
C	6.38102	-9.4789	-1.37941
C	8.70532	-8.71126	-1.41314
C	8.28484	-9.46559	1.28155
C	5.94831	-8.7347	1.3159
H	8.57827	1.34146	-0.06974
H	10.70499	-2.40803	-0.06692

H	9.47203	-4.51401	-0.06414
H	5.1611	-4.49307	-0.05307
H	4.80114	3.53862	-0.07622
H	6.93891	2.28983	-0.05881
H	3.53356	-3.52804	-0.15059
H	1.39745	-2.27692	-0.08746
H	-1.14004	4.50667	-0.1237
H	3.1658	4.48677	-0.34535
H	-0.20787	-1.32553	0.37932
H	-2.36117	2.40332	0.14686
H	-5.15611	-3.91632	1.4501
H	-5.69062	-3.09608	-0.08082
H	-4.0379	-3.84703	0.01908
H	-4.81556	-2.96756	2.93722
H	-3.34229	-1.91035	3.05538
H	-4.99486	-1.1591	2.95526
H	-7.20833	-0.54731	0.60343
H	-6.40685	-1.21557	2.09112
H	-6.26686	0.55443	1.70015
H	-6.60172	-0.95844	-1.03785
H	-5.02903	-0.28417	-1.64956
H	-5.16865	-2.05417	-1.25845
H	-0.98183	10.09728	-1.23011
H	0.55747	9.86914	-2.16857
H	-0.8692	8.78755	-2.48507
H	-1.06616	9.69758	0.5194
H	-1.04185	7.97139	1.08674
H	0.38506	9.05267	1.40318
H	2.94497	10.20138	-0.30918
H	1.40768	10.12048	0.65642
H	2.8471	9.12743	1.15371
H	3.03561	9.50523	-1.96386
H	3.03256	7.70663	-2.22311
H	1.5929	8.69939	-2.7203
H	13.59657	3.98996	0.30156
H	13.99766	3.01096	-1.17589
H	12.35905	3.78154	-1.01301
H	13.38657	3.20346	1.90312
H	11.92999	2.17615	2.25691
H	13.56843	1.40525	2.09385

H	15.57115	0.53481	-0.36305
H	14.90048	1.36033	1.11036
H	14.7289	-0.43951	0.91927
H	14.82471	0.77825	-1.97995
H	13.20571	0.05684	-2.38067
H	13.37693	1.8567	-2.18941
H	6.94739	-10.18356	-2.0286
H	5.93587	-10.03776	-0.52602
H	5.57091	-8.99818	-1.97221
H	8.65316	-9.62021	-2.05335
H	9.05344	-7.8479	-2.02321
H	9.4183	-8.88718	-0.57678
H	7.72973	-10.18028	1.92951
H	8.73895	-10.01587	0.4273
H	9.0871	-8.97297	1.87524
H	6.01443	-9.64372	1.95472
H	5.58652	-7.87784	1.92718
H	5.23847	-8.92043	0.479

Quaterrylene

C	3.30045	-2.40609	-0.00831
C	4.70965	-2.40819	-0.01651
C	5.44695	-1.23479	-0.02441
C	4.71845	-0.00139	-0.02481
C	3.29785	-0.00189	-0.01811
C	2.56445	-1.23089	-0.00931
C	5.44585	1.23271	-0.03051
C	-1.05125	2.40531	0.00549
C	3.29925	2.40271	-0.02791
C	2.56435	1.22691	-0.01811
C	6.91865	-1.24389	-0.02951
C	7.62825	0.00031	-0.03171
C	6.91745	1.24381	-0.03151
C	7.66425	-2.41609	-0.03081
C	9.08015	-2.41399	-0.03281
C	9.76545	-1.22469	-0.03361
C	9.04055	0.00151	-0.03311
C	9.76385	1.22881	-0.03291
C	9.07705	2.41721	-0.03091
C	7.66125	2.41711	-0.03011

C	1.09305	-1.23089	0.00269
C	1.09315	1.22701	-0.00571
C	0.35985	-0.00179	0.00839
C	-1.06075	-0.00109	0.02269
C	-1.78985	-1.23389	0.03699
C	-1.05265	-2.40739	0.02369
C	0.35635	-2.40589	0.00549
C	0.35775	2.40271	-0.00791
C	4.70825	2.40581	-0.03451
C	-1.78885	1.23221	0.01869
C	-3.26145	-1.24339	0.05569
C	-3.26035	1.24371	0.02209
C	-3.97145	0.00051	0.04239
C	-5.38385	0.00161	0.04579
C	-6.10835	-1.22469	0.06919
C	-5.42245	-2.41339	0.08969
C	-4.00675	-2.41539	0.08349
C	-4.00425	2.41671	0.00049
C	-5.41985	2.41671	-0.00151
C	-6.10695	1.22851	0.02159
H	2.76696	-3.36803	-0.00088
H	5.24102	-3.37134	-0.01665
H	-1.58231	3.36862	0.00547
H	2.76495	3.36422	-0.03062
H	7.13554	-3.3807	-0.03027
H	9.63126	-3.36598	-0.03373
H	10.86543	-1.21856	-0.03463
H	10.86384	1.22412	-0.03435
H	9.62681	3.36997	-0.02993
H	7.13105	3.3809	-0.02832
H	-1.58424	-3.37041	0.02746
H	0.88932	-3.36807	-0.00691
H	0.89164	3.36438	-0.02053
H	5.23911	3.36919	-0.04311
H	-7.20833	-1.21902	0.07081
H	-5.97322	-3.36534	0.11095
H	-3.47785	-3.37973	0.10132
H	-3.47412	3.3804	-0.01565
H	-5.96955	3.3693	-0.02159
H	-7.20694	1.22395	0.02141

QB4

C	6.01306	-2.41144	-0.01976
C	7.42226	-2.41354	-0.02796
C	8.15956	-1.24014	-0.03586
C	7.43106	-0.00674	-0.03626
C	6.01046	-0.00724	-0.02956
C	5.27706	-1.23624	-0.02076
C	8.15846	1.22736	-0.04196
C	1.66136	2.39996	-0.00596
C	6.01186	2.39736	-0.03936
C	5.27696	1.22156	-0.02956
C	9.63126	-1.24924	-0.04096
C	10.34086	-0.00504	-0.04316
C	9.63006	1.23846	-0.04296
C	10.37686	-2.42144	-0.04226
C	11.79276	-2.41934	-0.04426
C	12.47806	-1.23004	-0.04506
C	11.75316	-0.00384	-0.04456
C	12.47646	1.22346	-0.04436
C	11.78966	2.41186	-0.04236
C	10.37386	2.41176	-0.04156
C	3.80566	-1.23624	-0.00876
C	3.80576	1.22166	-0.01716
C	3.07246	-0.00714	-0.00306
C	1.65186	-0.00644	0.01125
C	0.92276	-1.23924	0.02555
C	1.65996	-2.41274	0.01225
C	3.06896	-2.41124	-0.00596
C	3.07036	2.39736	-0.01936
C	7.42086	2.40046	-0.04596
C	0.92376	1.22686	0.00725
C	-0.54884	-1.24874	0.04425
C	-0.54774	1.23836	0.01065
C	-1.25884	-0.00484	0.03095
C	-2.67124	-0.00374	0.03435
C	-3.39574	-1.23004	0.05775
C	-2.70984	-2.41874	0.07825
C	-1.29414	-2.42074	0.07205
C	-1.29164	2.41136	-0.01096

C	-2.70724	2.41136	-0.01296
C	-3.39434	1.22316	0.01015
B	12.58936	-3.7954	-0.04557
B	12.58432	3.78903	-0.04094
B	-3.5018	3.78829	-0.04197
B	-3.50596	-3.79474	0.10898
O	13.37124	-4.34385	-1.24476
O	12.67666	-4.74876	1.15278
O	13.41094	4.31469	-1.22015
O	12.62472	4.76643	1.14043
O	-5.03015	3.90785	-0.04655
O	-2.84001	5.17171	-0.06906
O	-2.84608	-5.17826	0.13345
O	-5.03521	-3.91254	0.11806
C	13.26032	-5.92651	0.5773
C	14.13022	-5.41187	-0.66089
C	14.33345	-6.53366	-1.67075
C	15.51454	-4.94941	-0.22578
C	14.14839	-6.63093	1.59448
C	12.19867	-6.92874	0.14375
C	13.65423	5.68774	0.7528
C	13.65858	5.67702	-0.8455
C	13.34153	7.08419	1.27404
C	15.00974	5.29272	1.32382
C	15.00971	6.13733	-1.37663
C	12.61029	6.62073	-1.41996
C	-3.94369	6.04411	-0.3515
C	-5.23541	5.30104	0.22643
C	-6.49316	5.80014	-0.4725
C	-5.42013	5.56412	1.71512
C	-3.74967	7.3874	0.33948
C	-4.07392	6.32533	-1.84263
C	-3.94426	-6.04256	0.45689
C	-5.24535	-5.31245	-0.11652
C	-3.76301	-7.40265	-0.20403
C	-4.04819	-6.28746	1.95648
C	-6.49091	-5.79303	0.6164
C	-5.45552	-5.61194	-1.59492
H	5.47957	-3.37338	-0.01232
H	7.95363	-3.37669	-0.02809

H	1.1303	3.36327	-0.00597
H	5.47755	3.35887	-0.04206
H	9.84815	-3.38605	-0.04171
H	13.57804	-1.22391	-0.04607
H	13.57645	1.21877	-0.04579
H	9.84366	3.37555	-0.03976
H	1.12837	-3.37576	0.01602
H	3.60193	-3.37342	-0.01835
H	3.60425	3.35903	-0.03197
H	7.95172	3.36384	-0.05455
H	-4.49572	-1.22437	0.05937
H	-0.76524	-3.38508	0.08988
H	-0.76151	3.37505	-0.02709
H	-4.49433	1.2186	0.00997
H	15.42276	-6.71	-1.81596
H	13.85476	-7.46433	-1.29197
H	13.87158	-6.24658	-2.64185
H	16.28955	-5.54735	-0.75551
H	15.64143	-3.87263	-0.47719
H	15.62426	-5.09023	0.87282
H	13.78724	-7.67297	1.74444
H	15.19614	-6.65232	1.21959
H	14.11082	-6.08254	2.56228
H	12.3559	-7.8916	0.67942
H	11.18943	-6.52878	0.38917
H	12.27479	-7.09818	-0.95364
H	14.17584	7.43426	1.92224
H	13.22176	7.78196	0.41525
H	12.39895	7.05687	1.86528
H	15.40051	6.1191	1.95878
H	14.89819	4.37281	1.94033
H	15.72078	5.09768	0.49006
H	14.86826	7.02605	-2.03157
H	15.67076	6.40767	-0.523
H	15.47726	5.31507	-1.9632
H	13.10736	7.38081	-2.06337
H	11.88213	6.03926	-2.02859
H	12.07556	7.13154	-0.58815
H	-7.19735	6.21626	0.2823
H	-6.22069	6.59475	-1.20264

H	-6.98035	4.95455	-1.00766
H	-6.40987	6.04305	1.88777
H	-5.37294	4.6006	2.27025
H	-4.61296	6.24063	2.07509
H	-3.75531	8.20043	-0.42061
H	-4.57669	7.55336	1.06561
H	-2.7757	7.39008	0.87813
H	-3.99335	7.42074	-2.02255
H	-3.26105	5.79902	-2.39127
H	-5.06206	5.96194	-2.20362
H	-3.75555	-8.19667	0.57587
H	-4.60279	-7.58579	-0.91114
H	-2.7987	-7.41944	-0.75955
H	-3.96484	-7.37824	2.16144
H	-3.22555	-5.74865	2.47773
H	-5.02965	-5.91465	2.32596
H	-7.20833	-6.22715	-0.11546
H	-6.20605	-6.56952	1.36116
H	-6.96835	-4.93403	1.13883
H	-6.44822	-6.09422	-1.73884
H	-5.41746	-4.66241	-2.17432
H	-4.65484	-6.29773	-1.95183
